# Machine Learning and UHPLC–MS/MS-Based Discrimination of the Geographical Origin of *Dendrobium officinale* from Yunnan, China

**DOI:** 10.3390/foods14193442

**Published:** 2025-10-08

**Authors:** Tao Lin, Yanping Ye, Jiao Zhang, Jing Wang, Zhengxu Hu, Khine Zar Linn, Xinglian Chen, Hongcheng Liu, Zhenhuan Liu, Qinghua Yao

**Affiliations:** 1 Quality Standards and Testing Technology Research Institute, Yunnan Academy of Agricultural Sciences, Kunming 650205, China; lintaonj@126.com (T.L.); 15987127177@163.com (Y.Y.); zj18285696956@163.com (J.Z.); chen544141152@163.com (X.C.); liuorg@163.com (H.L.); 2 Longling County Agricultural Technology Extension Center, Longling 678300, China; jingjingmichuer@163.com (J.W.); 18708755021@139.com (Z.H.); 3 Department of Biotechnology Research, Ministry of Science and Technology, Kyaukse 05021, Myanmar; khinezarlinn9@gmail.com; 4 Institute of Quality Standards Testing Technology for Agro-Products, Fujian Academy of Agricultural Sciences, Fuzhou 350003, China

**Keywords:** discrimination, *Dendrobium officinale*, geographical origin, machine learning, UHPLC–MS/MS

## Abstract

A rapid targeted screening method for 22 compounds, including flavonoids, glycosides, and phenolics, in *Dendrobium officinale* was developed using UHPLC–MS/MS, demonstrating good linear correlation coefficients, precision, repeatability, and stability. *D. officinale* from the Guangnan and Maguan regions can be effectively classified into two distinct categories using PCA. In addition, OPLS-DA discriminant analysis enables clear separation between groups, with samples forming well-defined clusters. The 22 chemical components provide valuable origin-related information for *D. officinale*. The compounds with VIP values of >1 included eriodictyol, vanillic acid, protocatechuic acid, gentisic acid, and naringenin. The difference in naringenin content between *D. officinale* from the two production areas was minimal. By contrast, eriodictyol and vanillic acid were relatively abundant in *D. officinale* from Guangnan, while gentisic acid and protocatechuic acid were more prevalent in *D. officinale* from Maguan. The pathways with higher Kyoto Encyclopedia of Genes and Genomes enrichment were primarily associated with lipid metabolism and atherosclerosis, fluid shear stress and atherosclerosis, and nonalcoholic fatty liver disease. These findings suggest that *D. officinale* exhibits promising lipid-balancing properties and potential cardiovascular health benefits. Seven machine learning algorithms—Random Forest, XGBoost, Support Vector Machine, k-Nearest Neighbor, Backpropagation Neural Network, Random Tree, and CatBoost—demonstrated superior accuracy and precision in distinguishing *D. officinale* from the Guangnan and Maguan regions. The key compounds with higher weights—vanillic acid, chrysoeriol, trigonelline, isoquercitrin, gallic acid, 4-hydroxybenzaldehyde, eriodictyol, sweroside, apigenin, and homoeriodictyol—play a crucial role in model construction and the identification of *D. officinale* from the Guangnan and Maguan regions. The quantification of 22 compounds using UHPLC–MS/MS, combined with PCA, OPLS-DA, and machine learning, enables effective discrimination of *D. officinale* from these two Yunnan production areas.

## 1. Introduction

*Dendrobium officinale*, a member of the orchid family *Dendrobium*, is one of China’s distinctive medicinal herbs. In November 2023, the National Health Commission and the State Administration of Market Supervision and Administration included *D. officinale* in the catalog of medicinal and food substances [1]. Owing to its notable health benefits—including antioxidant [2], anti-inflammatory [3], blood-sugar- and blood-pressure-lowering [4,5], liver-protective [6], and antitumor properties [7]—*D. officinale* has been widely used as a raw material in the food industry.

*D. officinale* is primarily distributed across China’s Anhui, Zhejiang, Guangxi, Yunnan, and other regions, with Yunnan Province serving as one of its key production areas [8]. Guangnan County, located in Wenshan City, Yunnan, is distinguished by its unique karst landscape and a subtropical plateau monsoon climate characterized by high humidity, significant temperature variations between day and night, and slightly alkaline soil. These environmental conditions contribute to the slow growth of *D. officinale* in the region, promoting the accumulation of bioactive compounds that enhance its medicinal value and health benefits. In recent years, this has made *D. officinale* highly sought after by consumers [9].

Guangnan *D. officinale* has strong brand effect and active function; however, its limited geographical range and production capacity make it difficult to meet the growing market demand. Driven by economic interests, some dishonest growers substitute cheaper, lower-quality *D. officinale* from outside the Guangnan region—such as Maguan County in Wenshan City—falsely presenting it as Guangnan *D. officinale*. As these counterfeit products are difficult to distinguish by appearance alone, they pose a significant challenge to the high-quality development and economic growth of the Guangnam *D. officinale* industry [8,10].

In recent years, various methods have been used for identifying *Dendrobium*, including near-infrared spectroscopy [11,12,13], stable isotopic and elemental analysis [14], nucleic acid technology [15,16], mass spectrometry [17,18,19], and NMR [20]. Near-infrared spectroscopy effectively distinguishes different *Dendrobium* species but requires complex data processing [8]. Stable isotopic and elemental analysis detects only specific elements; however, the necessary instruments—especially stable isotope mass spectrometers—are costly and demand rigorous sample pretreatment [21]. NMR technology faces challenges owing to the complexity of nuclear magnetic spectrum analysis and limited instrument sensitivity [20]. Nucleic acid technology relies on DNA probes, which pose certain limitations in practical applications [22]. Mass spectrometry typically employs nontargeted screening, achieving superior identification results. However, it generates large datasets, which increases the workload for data analysis [23,24]. Given these challenges, establishing a simple and effective identification method is crucial.

Machine learning overcomes the drawbacks of manual feature variable selection, which is inefficient and highly subjective. Not only can it analyze large datasets more efficiently and objectively, but it also significantly enhances the efficiency and accuracy of identification [25,26]. The integration of machine learning with mass spectrometry technology has made identification more effective, efficient, and accurate, and its applications have become increasingly widespread in recent years [27,28,29]. In most cases, machine learning is employed for the analysis and classification of non-targeted metabolomics data. Given the substantial volume of non-targeted metabolomics datasets, the overall data processing workload is also considerable. Targeted metabolomics is a qualitative and quantitative approach for analyzing specific target compounds. It reduces the workload of the screening process, enhances accuracy and sensitivity, and offers better specificity and stability. In recent years, it has been widely applied in tracing and identifying agricultural products [30,31,32]. To analyze flavonoids, glycosides, and phenolics in *D. officinale*, UHPLC–MS/MS was employed for the targeted analysis of samples cultivated in the Guangnan and Maguan regions of Wenshan City, Yunnan Province. By integrating chemometrics and machine learning, this study aims to establish a rapid and effective identification method for *D. officinale* from these production areas.

## 2. Materials and Methods

### 2.1. Reagents and Solutions

Apigenin, chrysoeriol, epicatechin gallate, eriodictyol, gallic acid, gentisic acid, homoeriodictyol, hyperoside, isoquercitrin, lonicerin, myricitrin, naringenin, naringin, protocatechuic acid, quercetin, schaftoside, scutellarein, sweroside, syringin, trigonelline, vanillic acid, and 4-hydroxybenzaldehyde (purity > 98%) were purchased from Shanghai Yuanye Bio-Technology Co., Ltd. (Shanghai, China). Methanol and acetonitrile (HPLC grade) were obtained from Merck KGaA (Darmstadt, Germany), while formic acid (mass spectrometry grade) was sourced from Dikma Technologies Inc. (Beijing, China). Ultrapure water was prepared using Elga’s water purification system (Wycombe, UK).

### 2.2. D. officinale Collection

**Figure 1:**
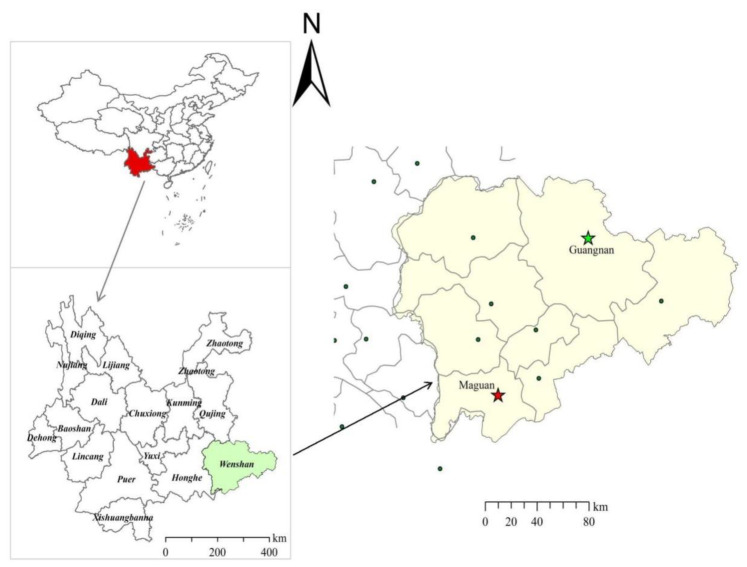
*D. officinale* collection area.

*D. officinale* samples were collected from Guangnan County and Maguan County, Wenshan City, Yunnan Province, China, as shown in Figure 1. A total of 45 *D. officinale* samples were obtained from each region, specifically from the aboveground stem parts of plants with a growth period of 3–4 years. Five branches were collected from each sample, with individual branches measuring approximately 15–20 cm in length. These were then cut into 2 cm segments, mixed, dried at 50 °C, crushed, and passed through a 0.28 μm sieve. Each sample was individually packaged in polyethylene bags and stored at 4 °C away from light.

### 2.3. Standard Solution Preparation

A total of 10 mg of each compound—apigenin, chrysoeriol, epicatechin gallate, eriodictyol, gallic acid, gentisic acid, homoeriodictyol, hyperoside, isoquercitrin, lonicerin, myricitrin, naringenin, naringin, protocatechuic acid, quercetin, schaftoside, scutellarein, sweroside, syringin, trigonelline, vanillic acid, 4-hydroxybenzoic acid, and 4-hydroxybenzaldehyde—was weighed. Each compound was dissolved in methanol, with the volume adjusted to 10 mL to prepare a 1 mg/mL standard solution. The solution was then transferred into 15 mL sealed brown reagent vials and stored at −4 °C away from light.

### 2.4. Sample Preparatison

A total of 2.5 g of *D. officinale* samples were weighed and mixed with 15 mL of aqueous methanol (8:2 ratio). The mixture was vortexed for 5 min, followed by sonication in a water bath for 30 min. This mixture was then centrifuged for 5 min, passed through a 0.22 μm filter membrane, and set aside for analysis.

### 2.5. UHPLC–MS/MS Parameters

The extracted *D. officinale* solution was injected into an AB QTRAP 5500 triple quadrupole mass spectrometer (Framingham, MA, USA) equipped with an ExionLC AD ultrahigh-performance liquid chromatograph (Framingham, MA, USA) and a Waters ACQUITY BEH C18 column (2.1 × 100 mm, 1.7 μm; Waters, Milford, MA, USA). The column temperature was maintained at 35 °C, with an injection volume of 2 μL. Gradient elution was performed using aqueous solution (A) containing 0.1% formic acid and 1 mmol/L ammonium acetate (B), acetonitrile (B), at a flow rate of 0.2 mL/min. The elution procedure was as follows: 0 min: 95% A, 3 min: 60% A, 5 min: 40% A, 8 min: 5% A, 10.2 min: 5% A, 10.3 min: 95% A, and 13 min: 95% A.

The mass spectrometry conditions were as follows: an ESI ion source operating in multiple reaction monitoring mode, with a spray voltage of 5500 V and an ion source temperature of 550 °C. The collision gas (CAD) was set to medium, with a gas flow rate of 20 L/h and a nebulizing gas flow rate of 55 L/h. Other mass spectrometry parameters are presented in Table 1.

### 2.6. Method Validation

The 22 compounds in *D. officinale* examined in this study were diluted to varying concentrations using methanol, and their linear regression equations were calculated by plotting the mass concentration of each compound on the *x*-axis and the corresponding peak area on the *y*-axis. The limit of detection (LOD) and limit of quantification (LOQ) were determined at *S*/*N* = 3 and *S*/*N* = 10, respectively. As the 22 compounds are natural products, the experiment was conducted with reference to method validation from the literature [55], focusing on precision, reproducibility, and stability. The precision test was conducted using the same *D. officinale* sample extraction solution, with the sample injected six consecutive times. The content of each compound was recorded, and its RSD value was calculated. For the repeatability test, the same *D. officinale* sample extraction solution was used to prepare six parallel samples, which were then injected and analyzed. The content of each compound was recorded, and the RSD for the 22 measured compounds was calculated. The stability test was conducted using the same *D. officinale* sample extraction solution, with compound content recorded at different time intervals—0, 2, 4, 8, 16, and 24 h—following injection and analysis. The RSD values were then determined.

### 2.7. Data Processing

The content of 22 compounds in *D. officinale* was analyzed by quantifying them using standard solutions. Their concentrations were further examined through principal component analysis (PCA), volcano plots, and heat maps. Model fitness and predictive performance were evaluated using R^2^(cum) and Q^2^(cum) through cross-validation. Representative differential metabolites were then selected for metabolic pathway enrichment analysis using metabolic pathway databases such as the Kyoto Encyclopedia of Genes and Genomes (KEGG, www.genome.jp/kegg, accessed on 7 May 2025).

SPSS software (v. 25) was used to perform machine learning on the dataset, prior to machine learning, the dataset was partitioned. The training and test sets were randomly divided in a 7:3 ratio, with the independent test set used for external validation of the model’s accuracy. To identify the optimal model for the machine learning evaluation of *D. officinale* origin identification data, various algorithms were employed, including Random Forest (RF), XGBoost, Support Vector Machine (SVM), k-Nearest Neighbor (KNN), Backpropagation Neural Network (BPNN), Random Tree (RT), and Catoost.

## 3. Results and Discussion

### 3.1. Optimization of Mass Spectrometry Conditions

All 22 compounds were diluted to 1 μg/mL using methanol and injected into the mass spectrometer via a syringe pump at a flow rate of 10 μL/min. Each compound was analyzed to identify precursor and product ions and to optimize DP and CE. The appropriate scanning mode was selected based on the peak profile of the target compound in the *D. officinale* sample. For example, although apigenin exhibits a strong response in the positive ion mode, an interference peak appears at the target peak position when analyzing the *D. officinale* sample. By contrast, the negative ion mode eliminates this interference at the target peak position, despite its response being approximately 10 times lower than that in the positive ion mode. Owing to the high apigenin content in the *D. officinale* samples, the use of the negative ion mode did not affect characterization or quantification, and no interference peaks were observed. The chromatograms of the 22 compounds are shown in Figure S1.

### 3.2. Method Validation

As presented in Table 2, the linear range was 0.001–15 μg/mL, with an *r*^2^ value of >0.999. The LOQ ranged from 0.006 to 1.2 mg/kg, while the LOD ranged from 0.001 to 0.4 mg/kg. The RSD values for precision, repeatability, and stability tests were 0.86–7.50%, 0.80–8.30%, and 1.40–8.38%, respectively. Results demonstrated that the 22 compounds exhibited good linear correlation coefficients, precision, repeatability, and stability, making them suitable for the determining flavonoids/glycosides, phenols, and other active compounds in *D. officinale*.

### 3.3. PCA and OPLS-DA for the Identification of D. officinale

**Figure 2:**
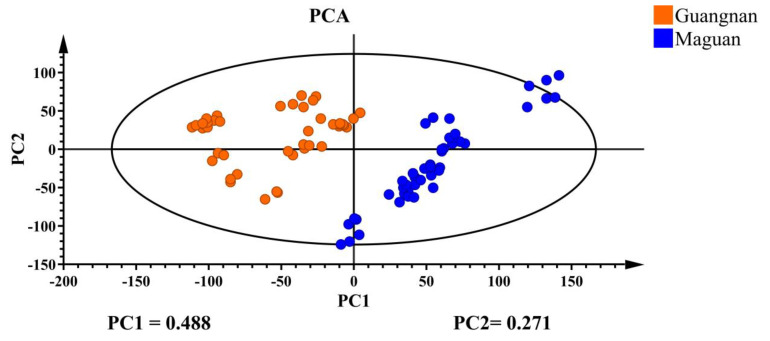
PCA plot of *D. officinale* from Guangnan and Maguan.

The 22 chemical components in 90 *D. officinale* samples were analyzed using unsupervised PCA, with the results shown in Figure 2. PCA effectively classified *D. officinale* from Guangnan and Maguan into two distinct categories. PC1 explained 48.8% of the variance and PC2 contributed 27.1%, resulting in a combined variance of 0.759, which meets the acceptable threshold in bioinformatics. This suggests that the 22 selected chemical constituents in this study can, to some extent, serve as representative markers for determining the origin of *D. officinale*.

**Figure 3:**
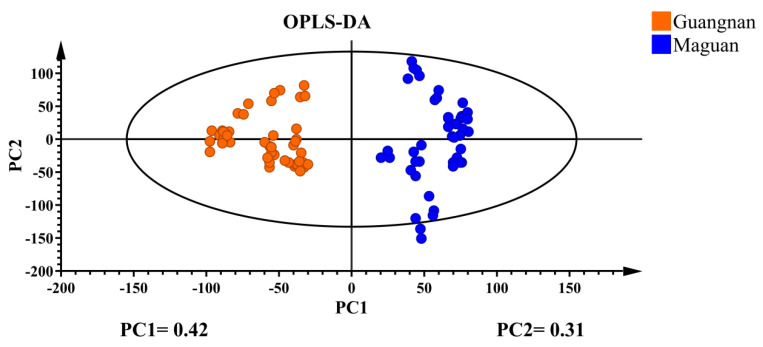
OPLS-DA plot of *D. officinale* from Guangnan and Maguan.

**Figure 4:**
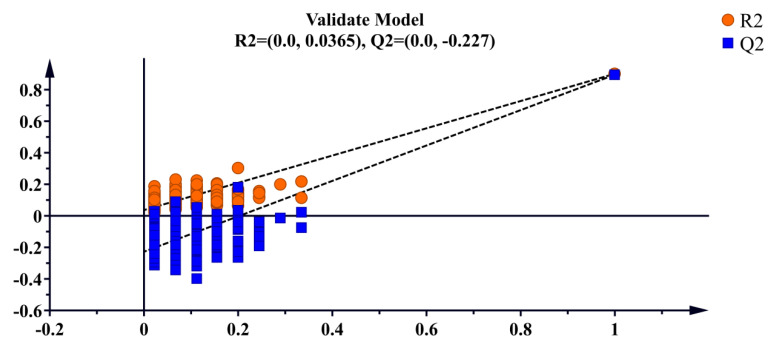
Cross-validation plot for the OPLS-DA model.

Supervised OPLS-DA discriminant analysis was applied to analyze the data, with the results shown in Figure 3. The *D. officinale* samples with different origins were clustered into distinct groups and were completely separable, showing significantly better clustering than PCA. This suggests that the supervised learning model effectively differentiates *D. officinale* from Guangnan and Maguan. Cross-validation results (Figure 4) indicate that R^2^ and Q^2^ values exceed 0.8, demonstrating a highly interpretable model with high predictability, confirming its validity for this analysis.

The variable importance point (VIP) values of the 22 compounds were obtained through OPLS-DA, where a higher VIP value indicates a greater contribution of a compound in differentiating *D. officinale* from Guangnan and Maguan. Compounds with high VIP values were classified as differential compounds, as shown in Figure S2.

**Figure 5:**
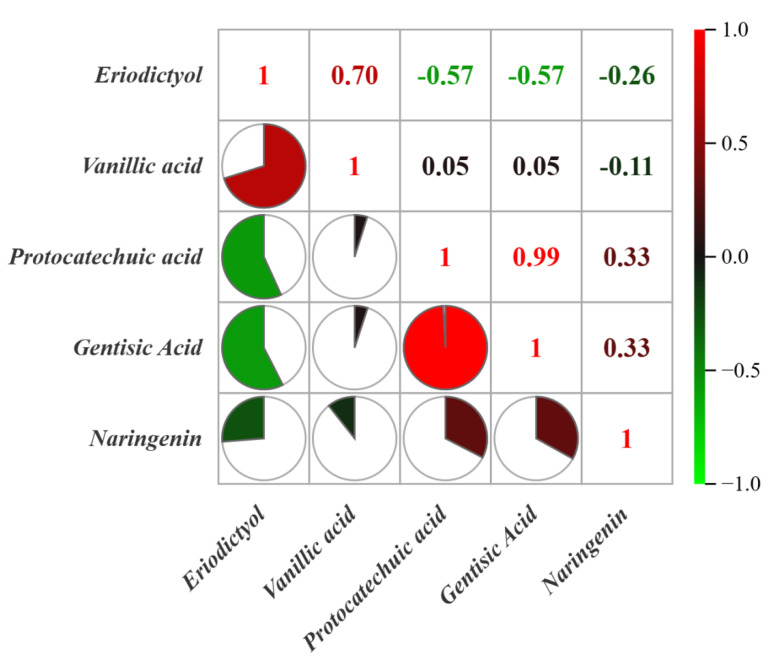
Correlation analysis of chemical compounds in *D. officinale* from Guangnan and Maguan.

The compounds with VIP values of >1 were flavonoids and phenolic acids, including eriodictyol, vanillic acid, protocatechuic acid, gentisic acid, and naringenin. This indicates that flavonoids and phenolic acids—precursor compounds in the biosynthetic pathway of phytosynthesis—exhibit significant variation under different environmental conditions. Differential compounds were subjected to correlation analysis, with results shown in Figure 5. The correlation coefficient between protocatechuic acid and gentisic acid was 0.99, suggesting that phenolic acids may undergo hydroxylation and decarboxylation transformations in plants, although further confirmation is required. In addition, the correlation coefficient between eriodictyol and vanillic acid was 0.70, indicating a possible pathway in which the methoxy (–OCH_3_) group in vanillic acid is converted to a hydroxyl (–OH) group within the plant body by demethylases, such as cytochrome P450 enzymes. The resulting protocatechuic acid can then enter the phenylpropanoid pathway, where it may be further transformed into p-coumaric acid or its derivatives, eventually leading to the synthesis of eriodictyol.

### 3.4. Heat Map and Volcano Plot Analyses for D. officinale Discrimination

**Figure 6:**
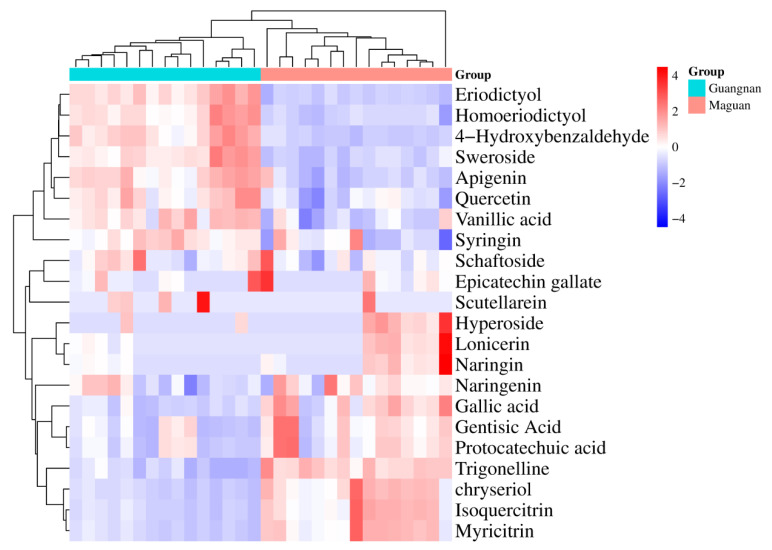
Heat map analysis of 22 compounds in *D. officinale* from Guangnan and Maguan.

The differences and correlations among the 22 compounds in *D. officinale* from Guangnan and Maguan were visualized using a heat map (Figure 6). Based on variations in compound content, the samples were clustered into two distinct groups, aligning with their geographical origins—Guangnan and Maguan. Overall, *D. officinale* from Guangnan contained higher levels of eriodictyol, homoeriodictyol, 4-hydroxybenzaldehyde, sweroside, apigenin, quercetin, vanillic acid, syringin, schaftoside, epicatechin gallate, and scutellarein. By contrast, samples from Maguan exhibited higher concentrations of hyperoside, lonicerin, naringin, naringenin, gallic acid, gentisic acid, protocatechuic acid, trigonelline, chrysoeriol, isoquercitrin, and myricitrin.

The results of the volcano plot analysis are shown in Figure S3. Among the 22 compounds, those with significant variations were eriodictyol, vanillic acid, protocatechuic acid, and gentisic acid, aligning with the findings of the VIP analysis. The violin plot (Figure S4) visualizes the differences in the content of five differential compounds in *D. officinale* from Guangnan and Maguan. The content of naringenin varied only slightly between the two regions, whereas eriodictyol and vanillic acid were relatively higher in *D. officinale* from Guangnan, while gentisic acid and protocatechuic acid had higher concentrations in samples from Maguan. These compounds may serve as active components distinguishing *D. officinale* from Guangnan and Maguan.

### 3.5. KEGG Pathway Analysis of Differential Metabolites in D. officinale from Guangnan and Maguan

**Figure 7:**
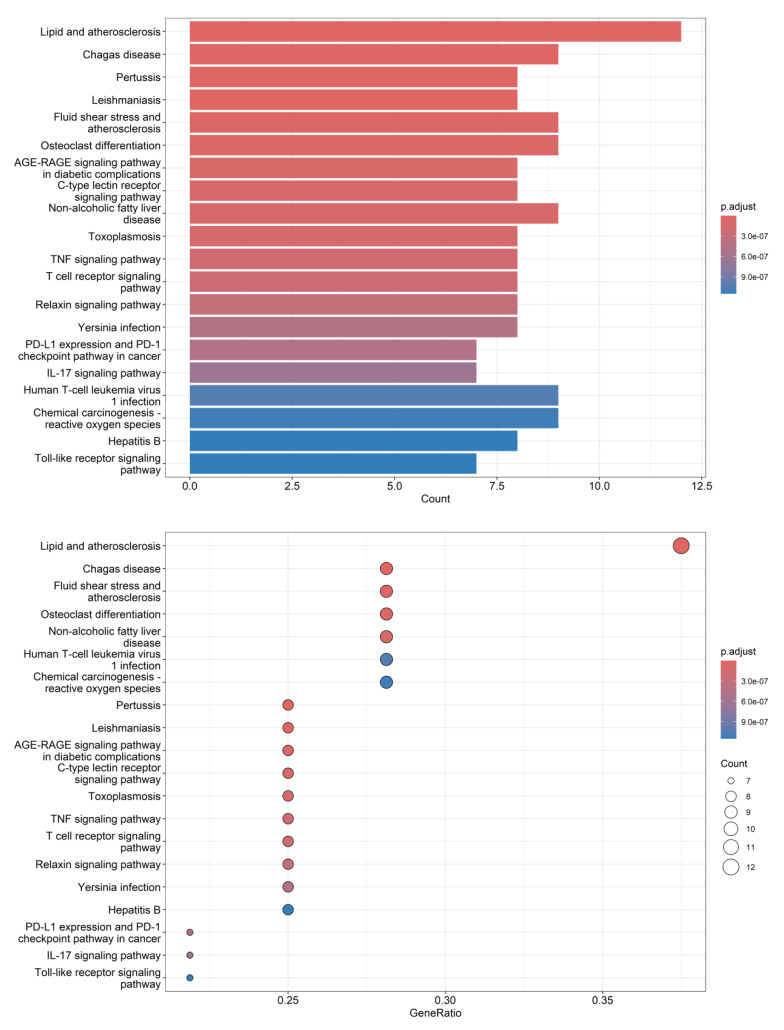
Bar plot and dot plot visualization of KEGG enrichment pathways for differential metabolites.

The differential compounds—eriodictyol, vanillic acid, protocatechuic acid, gentisic acid, and naringenin—with VIP values of >1 were entered into the BATMAN-TCM database (http://bionet.ncpsb.org/batman-tcm, accessed on 5 May 2025) to identify their potential action targets using a score cutoff of 0.86 as the screening criterion. KEGG signaling pathway enrichment analysis of the potential targets was then performed using the clusterProfiler module in the R software (v. 4.2.3) package. A threshold of *p* < 0.05 was applied to filter the relevant signaling pathways and visualize the enrichment results. A total of 136 signaling pathways were identified through screening, and the top 20 pathways were selected for visualization using bar and dot plots (Figure 7). The most enriched pathways were related to lipid metabolism and atherosclerosis, suggesting that *D. officinale* may offer health benefits for lipid homeostasis and cardiovascular health, which aligns with findings reported in the literature [19]. The biological activities of these five differential compounds have been documented in relevant literature. As an example, eriodictyol functions as a regulator of lipid metabolism [56,57], while vanillic acid offers protective effects against lipid-metabolizing enzymes [58]. In addition, protocatechuic acid, gentisic acid, and naringenin are known to alleviate atherosclerosis [59,60,61,62,63] and may serve as key active compounds in the lipid and atherosclerosis pathway. Furthermore, enrichment analysis highlighted significant involvement in the fluid shear stress and atherosclerosis pathway as well as the nonalcoholic fatty liver disease pathway, reinforcing the potential cardiovascular protective effects of *D. officinale*. These findings provide valuable insight into the development of *Dendrobium* resources in Yunnan Province, China. However, in this experiment, the KEGG pathways of the compounds already identified cannot fully substantiate their biological effects. Further analysis is required to examine the correlation between the metabolic degradation levels of these compounds and their potential effect concentrations, which may necessitate subsequent investigations.

### 3.6. Machine Learning

Machine learning, a generalized linear regression analysis model, effectively handles a large number of input features and is used for binary classification problems, making it a powerful tool in supervised learning [64,65]. In the experiments, accuracy, precision, recall, and F1 score were used to evaluate and compare the performance of different machine learning models in classifying training and testing sets of *D. officinale* samples from Guangnan and Maguan. Higher accuracy indicates better model performance in origin prediction. Precision represents the proportion of correctly predicted samples, with a higher precision reflecting the ability of the model to accurately identify the origin. Recall measures the percentage of correct predictions made by the origin identification model, with a higher recall indicating improved recognition of samples from different origins. The F1 score is the harmonic mean of precision and recall, ranging from 0 to 1. The closer the F1 score is to 1, the better the overall performance of the model. Results are presented in Table 3, showing that the accuracy and precision of the seven machine learning models were relatively high, which may be related to the significant variations in the content of the selected variables across different *D. officinale* from Guangnan and Maguan.

Among various machine learning methods, SVM and KNN are sensitive to feature scale and require Z-score standardization or normalization of data. In this experiment, both processed and unprocessed data achieved 100% prediction accuracy. Feature scaling had minimal impact on prediction results for RF, RT, XGBoost, BPNN, and CatBoost. Regardless of data processing, the prediction accuracy for the target variable class was 100%. To maintain consistency in the workflow, the “none” processing method was selected for all machine learning models used in this study. Other studies have also demonstrated that machine learning models such as RF [66], XGBoost [67], SVM [68], and KNN [69] exhibit excellent accuracy and precision, consistent with the findings of this research. The result indicates a significant difference in the chemical composition of *D. officinale* between the two origins. In addition, the application of various machine learning models effectively enhances the identification of *D. officinale* from Guangnan and Maguan.

The 22 measured compounds were modeled as independent variables, while class was modeled as the dependent variable. Compounds with weight values of >0 were selected for feature weight plots. As shown in Figure S5, the compounds with higher weights primarily included vanillic acid, chrysoeriol, trigonelline, isoquercitrin, gallic acid, 4-hydroxybenzaldehyde, eriodictyol, sweroside, apigenin, and homoeriodictyol. These compounds played a key role in model construction, suggesting they are critical for distinguishing *D. officinale* from Guangnan and Maguan. It also demonstrates that the flavonoids/glycosides and phenolic compounds selected from the *D. officinale* in the experiment were representative and can effectively trace the origin of *D. officinale* from Guangnan and Maguan with machine learning.

## 4. Conclusions

A rapid target screening method for 22 compounds, including flavonoids/glycosides and phenols, in *D. officinale* was developed using UHPLC–MS/MS. The linear ranges of the 22 compounds were 0.001–15 μg/mL, with *r*^2^ of >0.999. The LOQs ranged from 0.006 to 1.2 mg/kg, while the LODs ranged from 0.001 to 0.4 mg/kg. The RSD values were as follows: precision, 0.86–7.50%; repeatability, 0.80–8.30%; and stability, 1.40–8.38%, demonstrating strong reliability across tests. These results confirm that the 22 compounds demonstrated strong linear correlation coefficients, along with high precision, repeatability, and stability, enabling rapid and accurate screening of active compounds such as flavonoids/glycosides and phenols in *D. officinale*.

PCA effectively classified *D. officinale* from Guangnan and Maguan into two distinct categories, with PC1 and PC2 accounting for a combined variance of 0.759. Through OPLS-DA discriminant analysis, the samples exhibited intragroup aggregation and were completely separable between groups, demonstrating significantly better clustering than PCA. This indicates that the supervised learning model can effectively distinguish *D. officinale* from Guangnan and Maguan. Cross-validation results suggest that the model is highly interpretable and predictive, and the 22 chemical components serve as informative markers for determining the origin of *D. officinale*.

The compounds with VIP values of >1 include eriodictyol, vanillic acid, protocatechuic acid, gentisic acid, and naringenin. The correlation coefficient between protocatechuic acid and gentisic acid was 0.99, while the correlation coefficient between eriodictyol and vanillic acid was 0.70. Heat map analysis revealed that the 22 compounds could be clustered into two distinct groups based on differences in their content, aligning with the distribution of the two origins—Guangnan and Maguan. Volcano plot analysis identified eriodictyol, vanillic acid, protocatechuic acid, and gentisic acid as the compounds with significant variations, consistent with the VIP analysis results. The content of naringenin showed minimal differences between the two production areas. Overall, eriodictyol and vanillic acid were relatively high in *D. officinale* from Guangnan, while gentisic acid and protocatechuic acid were more abundant in samples from Maguan.

The KEGG signaling pathway enrichment analysis revealed that the most enriched pathways were related to lipid metabolism and atherosclerosis. In addition, significant enrichment was observed in fluid shear stress and atherosclerosis, nonalcoholic fatty liver disease, and other pathways, highlighting the potential health benefits of *D. officinale* in lipid homeostasis and cardiovascular health.

The seven machine learning models—RF, XGBoost, SVM, KNN, BPNN, RT, and CatBoost—demonstrated higher accuracy and precision in classification. The compounds with higher weights, including vanillic acid, chrysoeriol, trigonelline, isoquercitrin, gallic acid, 4-hydroxybenzaldehyde, eriodictyol, sweroside, apigenin, and homoeriodictyol, played a key role in the model construction and identification of *D. officinale* from Guangnan and Maguan.

## Figures and Tables

**Table 1:** Mass spectrometry parameters of 22 active compounds.

Compound	Precursor Ion (Da)	Product Ion (Da)	Declustering Potential (V)	Collision Energy (V)	References
Apigenin	269.1	117.1	−55	−55	[33]
Chrysoeriol	299.1	284.2	−45	−55	[34]
Epicatechin gallate	441.0	169.1	−50	−40	[35]
Eriodictyol	286.9	135.0	−50	−25	[36]
Gallic acid	169.0	125.1	−70	−30	[37]
Gentisic acid	153.0	108.1	−30	−45	[38]
Homoeriodictyol	301.1	150.8	−28	−45	[39]
Hyperoside	463.2	300.0	−165	−30	[40]
Isoquercitrin	463.1	300.1	−170	−30	[41]
Lonicerin	593.0	284.0	−190	−45	[42]
Myricitrin	463.0	271.1	−80	−55	[43]
Naringenin	271.1	150.9	−80	−30	[44]
Naringin	579.1	271.1	−210	−45	[45]
Protocatechuic acid	153.1	109.2	−80	−25	[46]
Quercetin	301.0	151.1	−100	−35	[47]
Schaftoside	563.1	353.0	−100	−55	[48]
Scutellarein	461.0	285.1	−30	−25	[49]
Sweroside	357.0	125.1	−120	−25	[50]
Syringin	394.9	232.1	120	40	[51]
Trigonelline	138.1	94.1	15	40	[52]
Vanillic acid	167.1	152.2	−45	−25	[53]
4-Hydroxybenzaldehyde	121.0	92.1	−40	−30	[54]

**Table 2:** Analytical parameters for each of the 22 compounds: linear range, correlation coefficient, LOD, LOQ, accuracy, and precision.

Compound	Linear Range (μg/mL)	Correlation Coefficient (*r*^2^)	Limit of Quantification (mg/kg)	Limit of Detection (mg/kg)	RSD/%		
					Precision	Repeatability	Stability
Apigenin	0.01–15	0.9993	0.06	0.020	2.34	2.36	2.43
Chrysoeriol	0.005–15	0.9993	0.03	0.001	6.84	8.30	6.82
Epicatechin gallate	0.002–15	0.9994	0.012	0.004	3.46	2.74	2.24
Eriodictyol	0.02–15	0.9991	0.12	0.040	2.45	3.46	4.75
Gallic acid	0.005–15	0.999	0.03	0.010	2.98	1.47	1.76
Gentisic acid	0.08–15	0.9995	0.48	0.150	2.48	4.26	3.92
Homoeriodictyol	0.008–15	0.9997	0.048	0.015	2.66	5.58	4.31
Hyperoside	0.01–15	0.9993	0.06	0.020	3.15	5.36	5.25
Isoquercitrin	0.005–15	0.9992	0.03	0.010	7.50	6.43	7.81
Lonicerin	0.002–15	0.9995	0.012	0.005	1.84	1.19	1.40
Myricitrin	0.005–15	0.9997	0.03	0.010	1.92	1.40	2.22
Naringenin	0.005–15	0.9993	0.03	0.010	0.86	0.80	1.58
Naringin	0.001–15	0.9998	0.006	0.002	3.87	4.61	4.92
Protocatechuic acid	0.08–15	0.9992	0.48	0.150	2.88	2.71	5.76
Quercetin	0.005–15	0.9994	0.03	0.010	6.44	8.19	6.82
Schaftoside	0.005–15	0.9993	0.03	0.010	1.48	1.70	2.30
Scutellarein	0.002–15	0.9995	0.012	0.004	2.93	3.14	4.62
Sweroside	0.005–15	0.9998	0.03	0.010	5.63	6.04	6.58
Syringin	0.001–15	0.999	0.006	0.002	5.18	6.01	8.38
Trigonelline	0.08–15	0.9991	0.48	0.150	0.97	1.25	2.43
Vanillic acid	0.2–15	0.9993	1.2	0.400	0.87	2.58	3.51
4-Hydroxybenzaldehyde	0.1–15	0.9994	0.6	0.200	1.23	1.31	1.92

**Table 3:** Comparison of classification accuracy across different machine learning models.

Arithmetic	Accuracy (%)	Precision (%)	Recall (%)	F1 Score
Random Forest (RF)	100	100	100	1
XGBoost	100	100	100	1
Support Vector Machine (SVM)	100	100	100	1
k-Nearest Neighbor (KNN)	100	100	100	1
Backpropagation Neural Network (BPNN)	100	100	100	1
Random Tree (RT)	100	100	100	1
CatBoost (CT)	100	100	100	1

## Data Availability

The original contributions presented in this study are included in the article/Supplementary Material. Further inquiries can be directed to the corresponding authors.

## Supplementary Materials

The following supporting information can be downloaded at: https://www.mdpi.com/article/10.3390/foods14193442/s1, Figure S1: Chromatograms of 22 compounds; Figure S2: VIP plots for OPLS-DA-based discrimination of D. officinale from Guangnan and Maguan; Figure S3: Volcano plot analysis of 22 compounds in D. officinale from Guangnan and Maguan; Figure S4: Violin plot analysis of differential metabolites in D. officinale from Guangnan and Maguan; Figure S5: Feature weight plot for different machine learning models.
