# Supplementary figures and images for: Machine Learning and UHPLC–MS/MS-Based Discrimination of the Geographical Origin of Dendrobium officinale from Yunnan, China
Source: Foods. 2025 Oct 8;14(19):3442. doi: 10.3390/foods14193442 (PMC12523558; doi:10.3390/foods14193442)

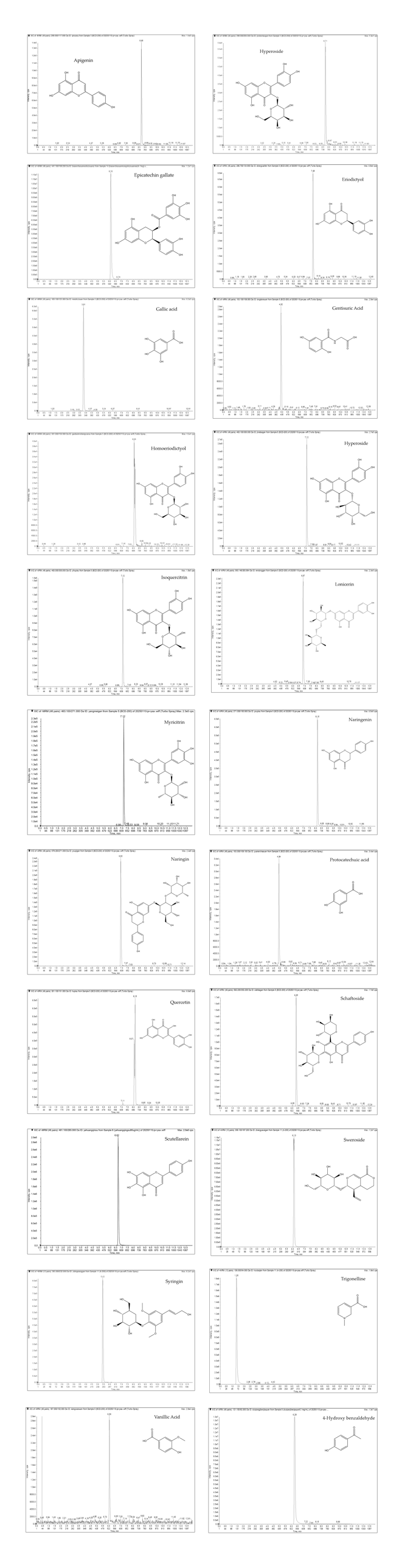

Supplement: Supplementary file 1 [file foods-14-03442-s001.zip › Figure S1.jpg]

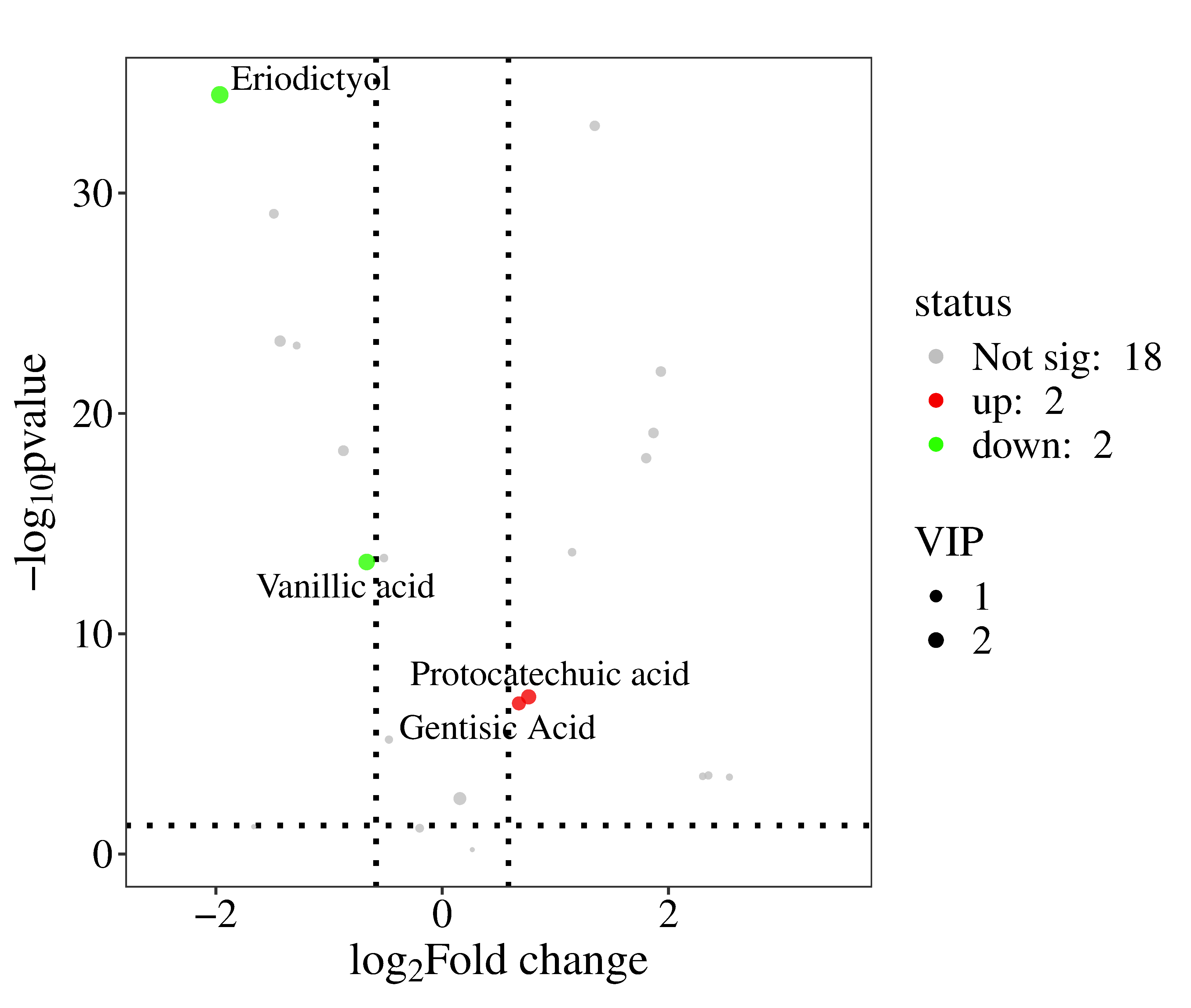

Supplement: Supplementary file 1 [file foods-14-03442-s001.zip › Figure S3.tiff]

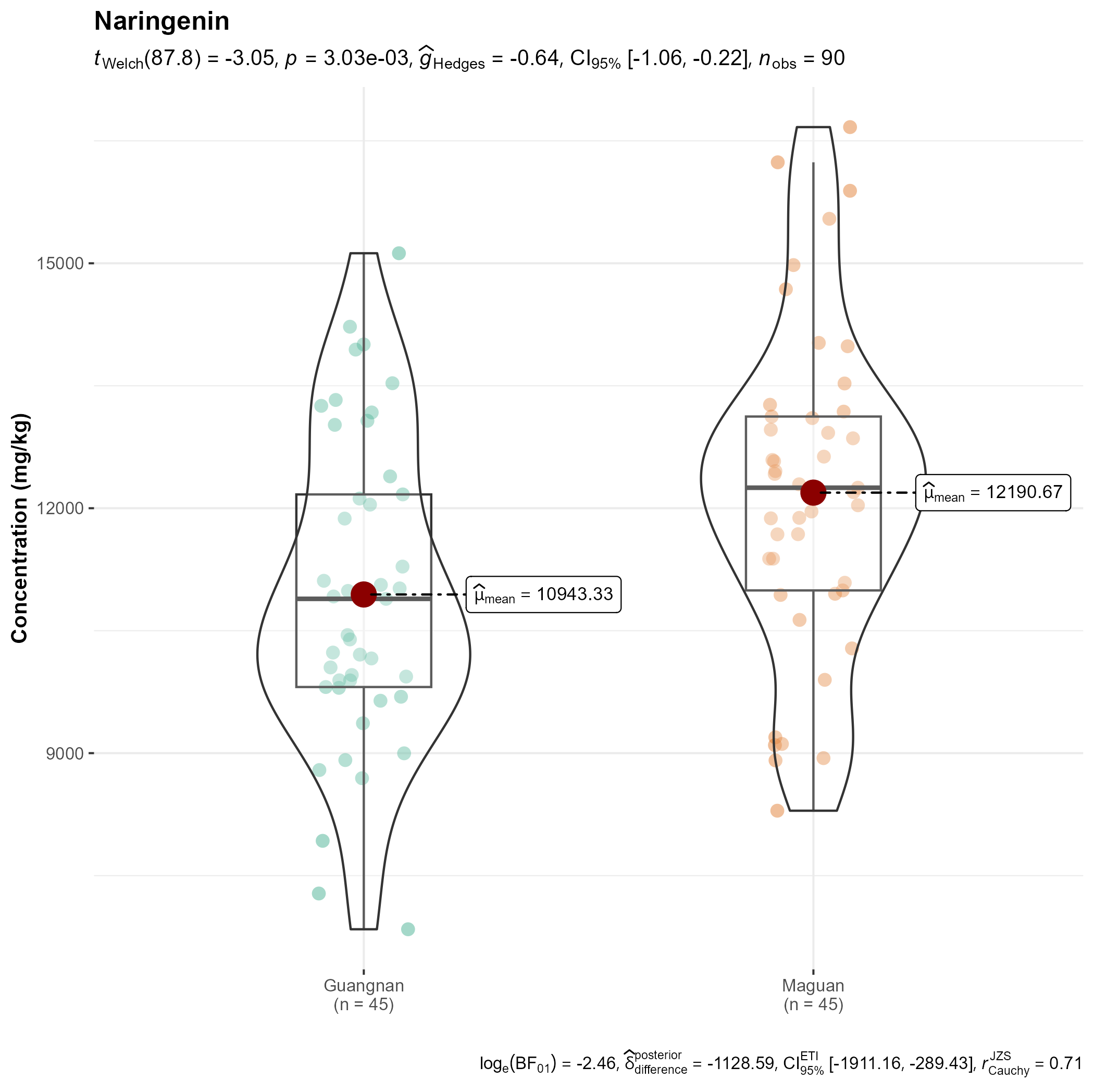

Supplement: Supplementary file 1 [file foods-14-03442-s001.zip › Figure S4-1.tiff]

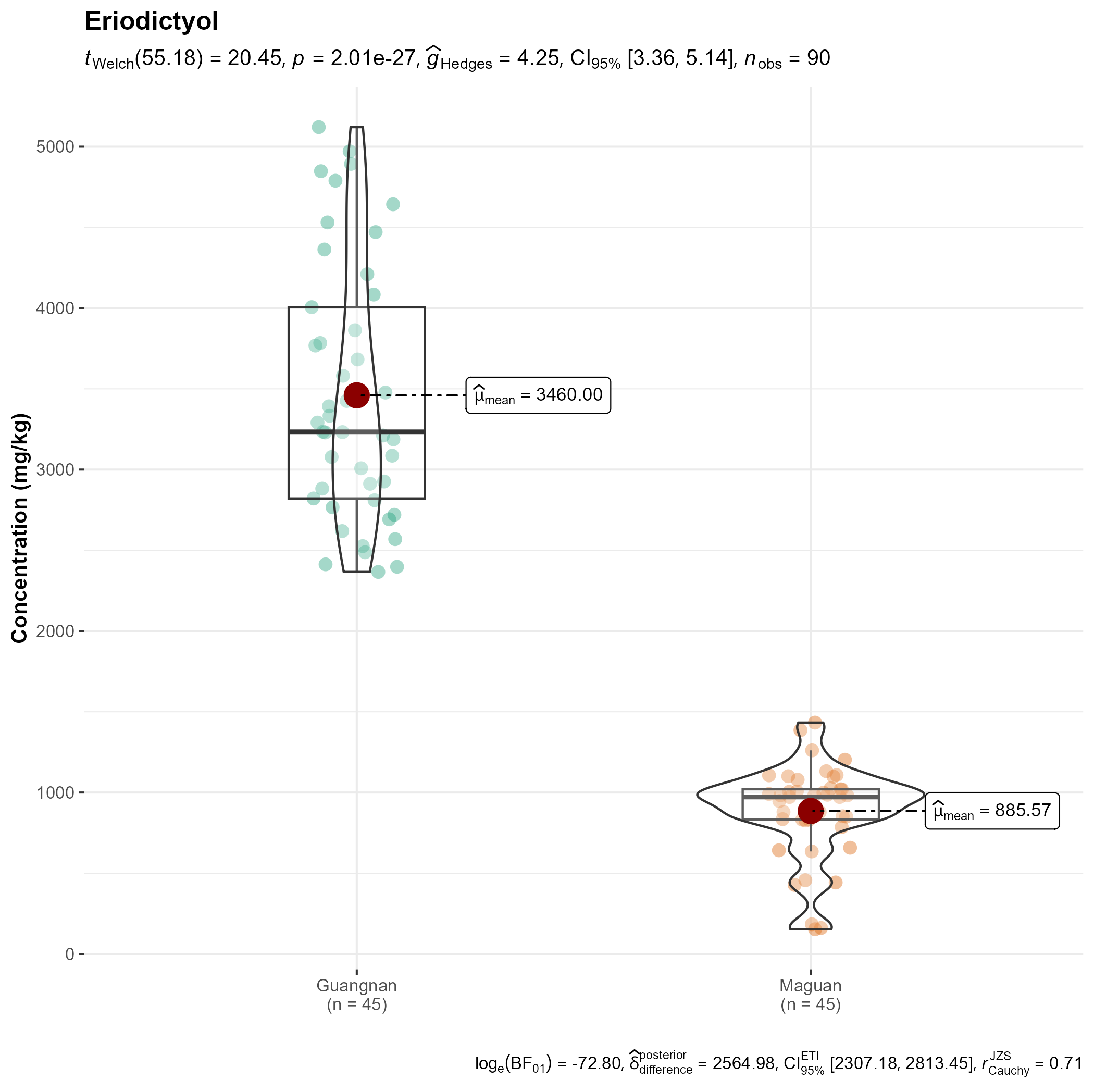

Supplement: Supplementary file 1 [file foods-14-03442-s001.zip › Figure S4-2.tiff]

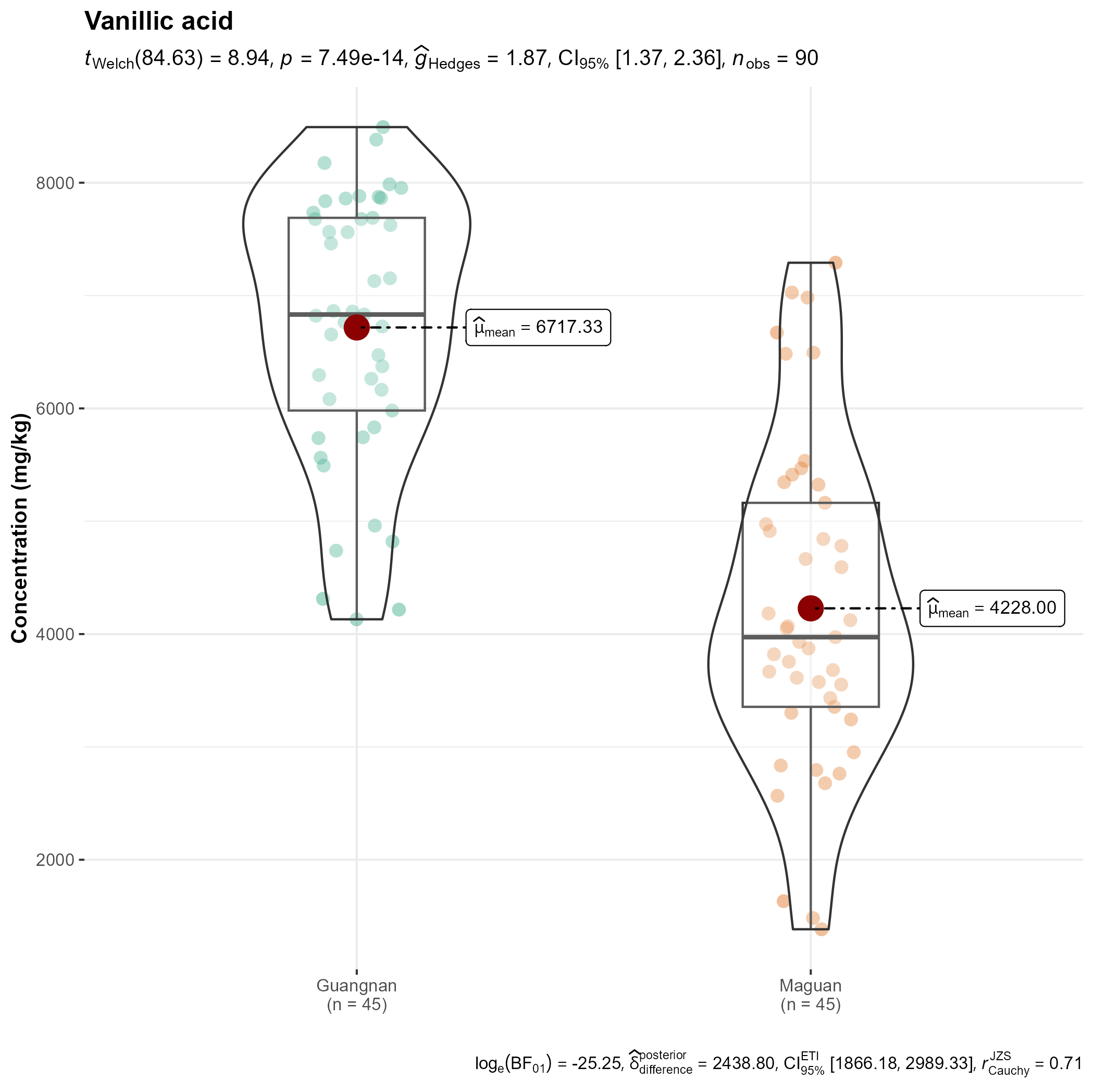

Supplement: Supplementary file 1 [file foods-14-03442-s001.zip › Figure S4-3.tiff]

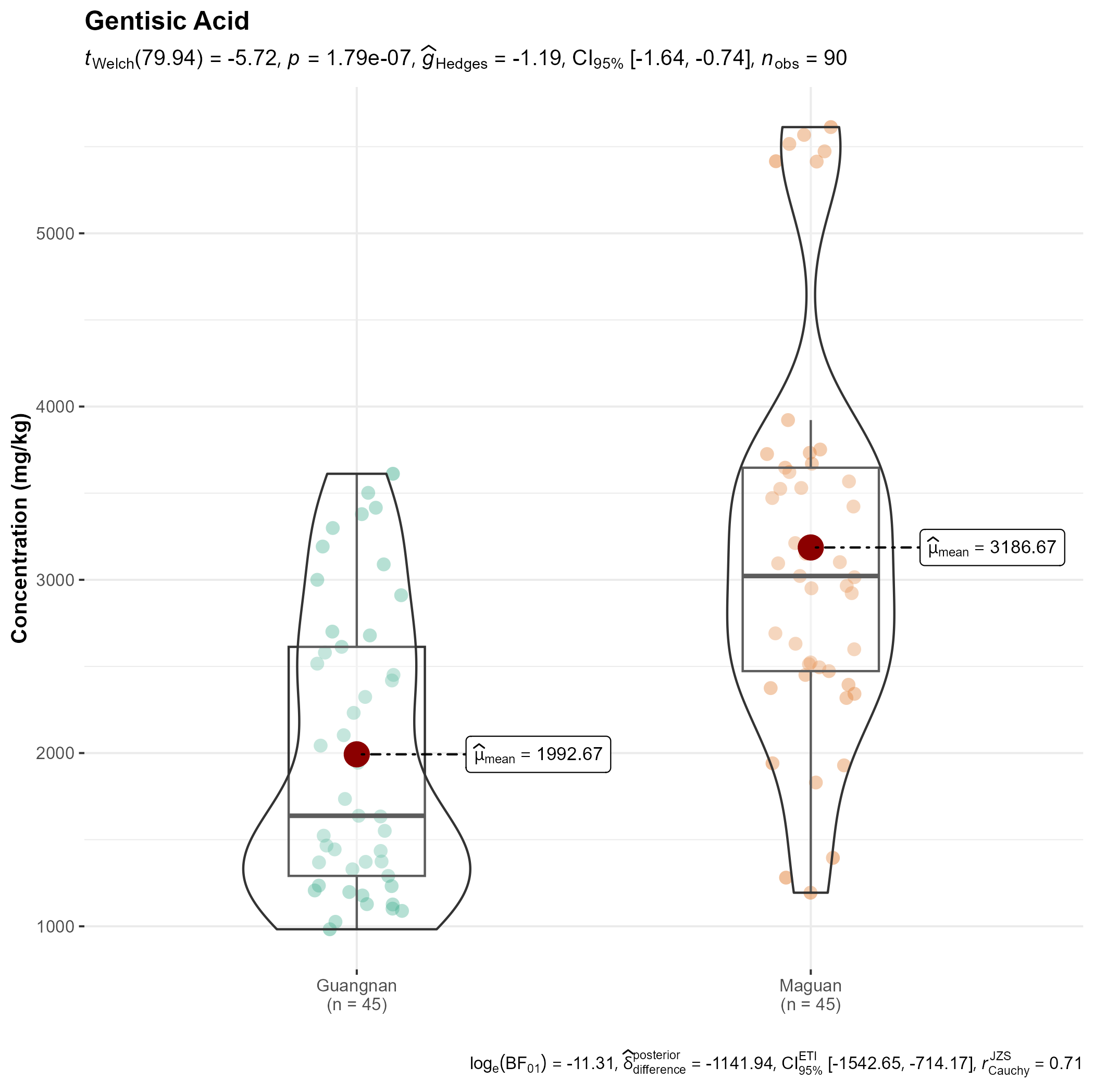

Supplement: Supplementary file 1 [file foods-14-03442-s001.zip › Figure S4-4.tiff]

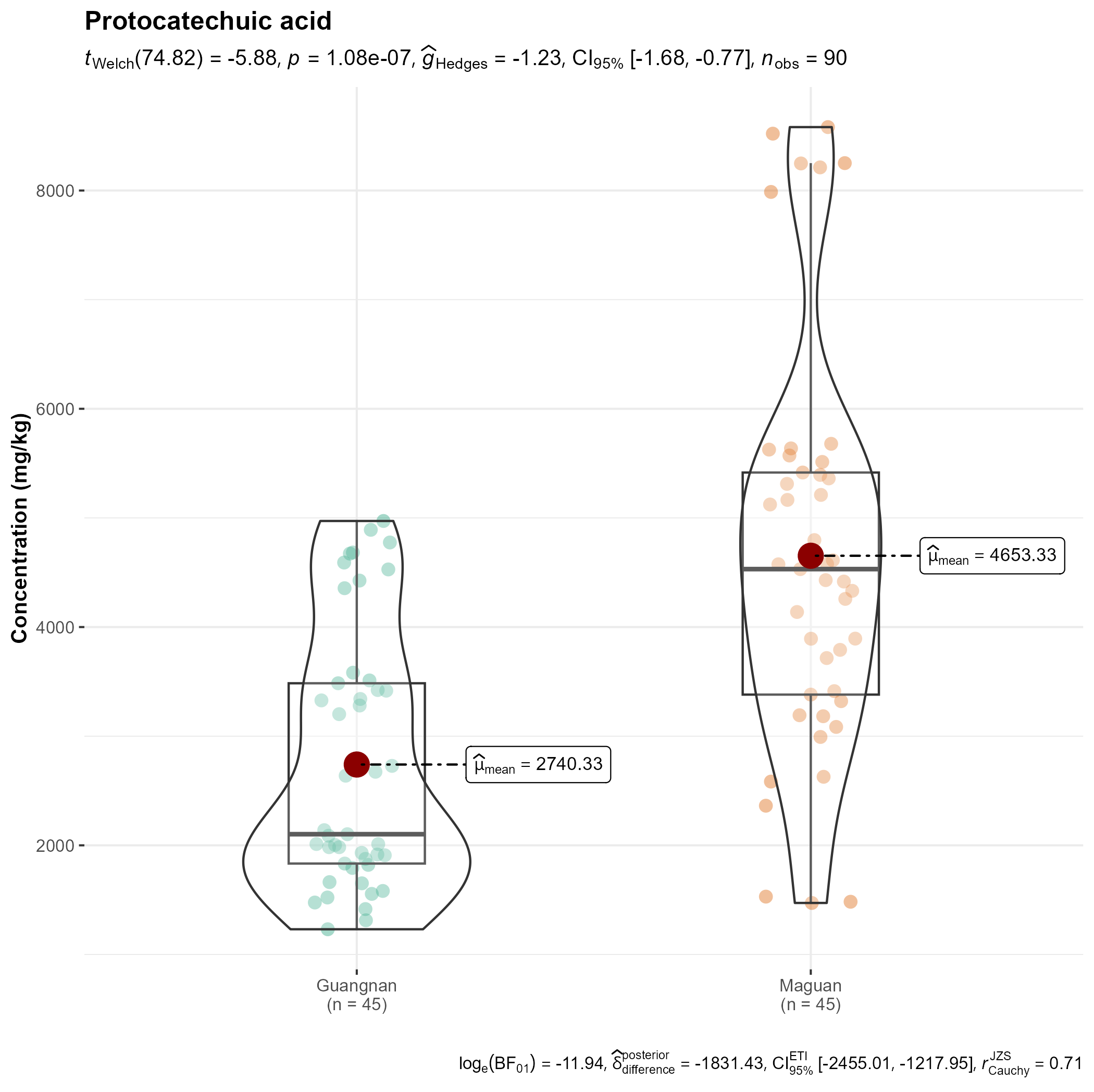

Supplement: Supplementary file 1 [file foods-14-03442-s001.zip › Figure S4-5.tiff]

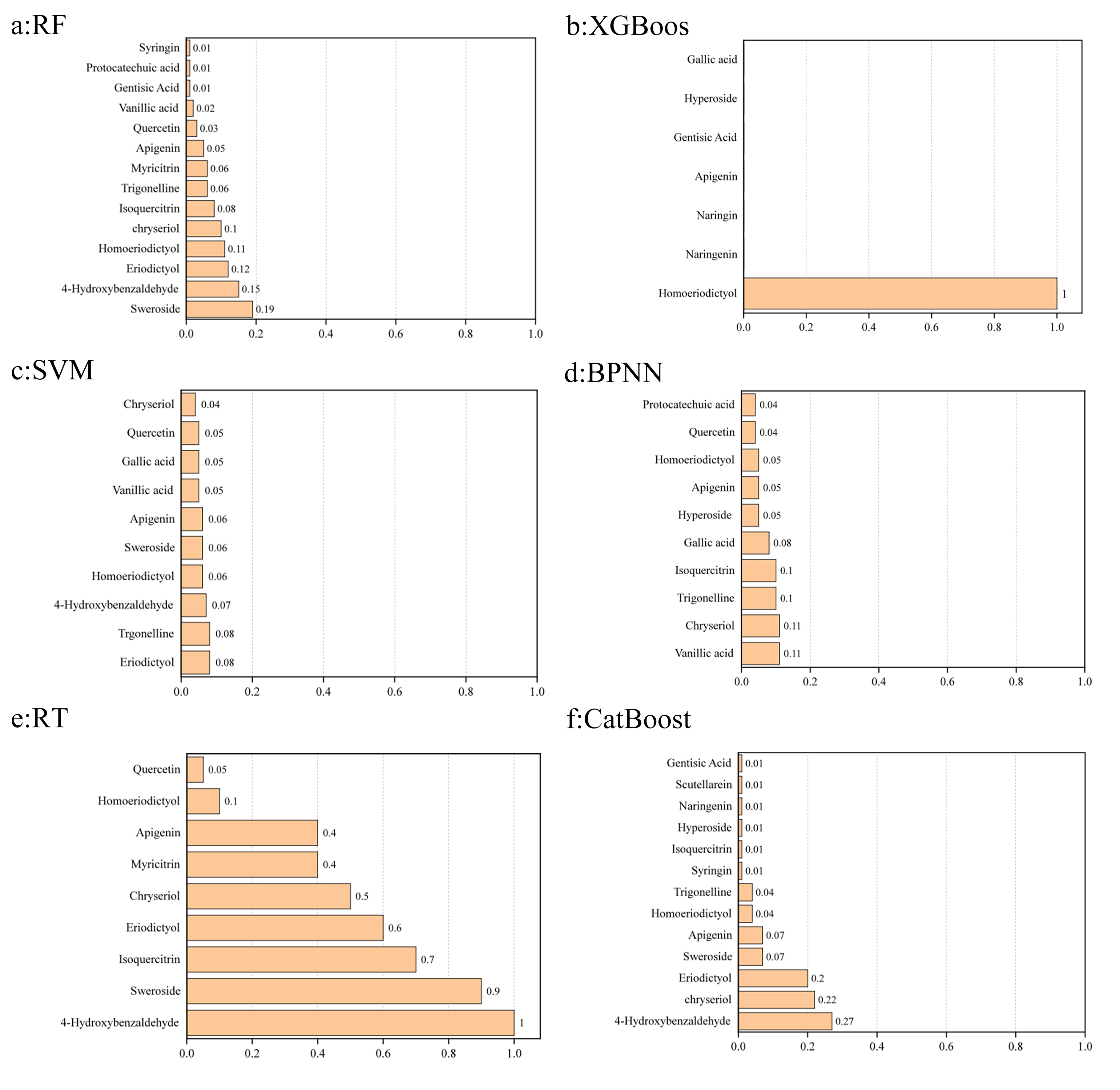

Supplement: Supplementary file 1 [file foods-14-03442-s001.zip › Figure S5.jpg]
